# Continuous glucose monitors prove highly accurate in critically ill children

**DOI:** 10.1186/cc9280

**Published:** 2010-10-06

**Authors:** Brian C Bridges, Catherine M Preissig, Kevin O Maher, Mark R Rigby

**Affiliations:** 1 Department of Pediatrics, Division of Pediatric Critical Care, Vanderbilt University School of Medicine, 2200 Children's Way, Nashville, TN 37232-9075, USA; 2 Department of Pediatrics, Emory University School of Medicine, 1405 Clifton Road NE, Atlanta, GA 30322-1060, USA; 3 Pediatric Critical Care, Children's Healthcare of Atlanta at Egleston, 1405 Clifton Road NE, Atlanta, GA 30322-1060, USA; 4 Pediatric Critical Care, The Children's Hospital at the Medical Center of Central Georgia, 777 Hemlock Street, Macon, GA 31201-2155, USA; 5 Pediatric Cardiac Critical Care, Sibley Heart Center Cardiology, 1405 Clifton Road NE, Atlanta, GA 30322-1060, USA

## Abstract

**Introduction:**

Hyperglycemia is associated with increased morbidity and mortality in critically ill patients and strict glycemic control has become standard care for adults. Recent studies have questioned the optimal targets for such management and reported increased rates of iatrogenic hypoglycemia in both critically ill children and adults. The ability to provide accurate, real-time continuous glucose monitoring would improve the efficacy and safety of this practice in critically ill patients. The aim of our study is to determine if a continuous, interstitial glucose monitor will correlate with blood glucose values in critically ill children.

**Methods:**

We evaluated 50 critically ill children age 6 weeks to 16 years old with a commercially available continuous glucose monitor (CGM; Medtronic Guardian^®^). CGM values and standard blood glucose (BG) values were compared. During the study, no changes in patient management were made based on CGM readings alone.

**Results:**

Forty-seven patients had analyzable CGM data. A total of 1,555 CGM and routine BG measurements were compared using Clarke error grid and Bland-Altman analysis. For all readings, 97.9% were within clinically acceptable agreement. The mean absolute relative difference between CGM and BG readings was 15.3%. For the 1,555 paired CGM and BG measurements, there is a statistically significant linear relationship between CGM values and BG (*P *<.0001). A high degree of clinical agreement existed in three subpopulation analyses based on age, illness severity, and support measures. This included some of our smallest patients (that is, <12 months old), those who required vasopressors, and those who were treated for critical illness hyperglycemia.

**Conclusions:**

In one of the largest studies to date, in a highly vulnerable ICU population, CGM values have a clinically acceptable correlation with the BG values now used diagnostically and therapeutically. Our data contest the theoretical concerns posed by some regarding CGM use in the ICU. The existing medical evidence may now support a role for CGM devices in the identification and management of hyperglycemia in diverse ICU settings.

## Introduction

Hyperglycemia is a risk factor for morbidity and mortality in critical illness. Active glycemic control with insulin can improve outcomes. This has been demonstrated in a variety of adult settings and recently in a mixed medical/surgical pediatric intensive care unit (ICU) [1-4]. The most substantive drawback to aggressive glycemic control in ICUs is iatrogenic hypoglycemia. Several recent, large multi-center randomized controlled trials (RCTs), including the Glucontrol, VISEP, and NICE-SUGAR trials, have been plagued with unacceptably high rates of hypoglycemia in strict control arms [5-7]. This resulted in premature study closure in some of these trials. In the first pediatric ICU glycemic RCT, published in the *Lancet *in February of 2009, the rate of hypoglycemia was 25% in the strict control arm group [4]. Concerns regarding hypoglycemia, substantiated by such trials, have caused major medical oversight committees to recommend a less strict approach to glycemic control [8-10]. Therapy-induced hypoglycemia is the primary reason many pediatric intensivists are reluctant to adopt standard glycemic control approaches, likely due to the potential adverse effects of low BG levels on the developing brain [11,12].

Both those who support or challenge glycemic control efforts in critical care settings agree that glycemic management could be made significantly safer and more efficient if there existed a means to more frequently and reliably track patients' glucose levels. Within the past decade, continuous glucose measurement devices have been developed and approved to assist with outpatient diabetes management. Due to concerns regarding altered perfusion in critical illness, many have questioned the accuracy of such devices in ICUs.

## Materials and methods

### Study design, patient enrollment, CGM placement and monitoring

**Figure 1:**
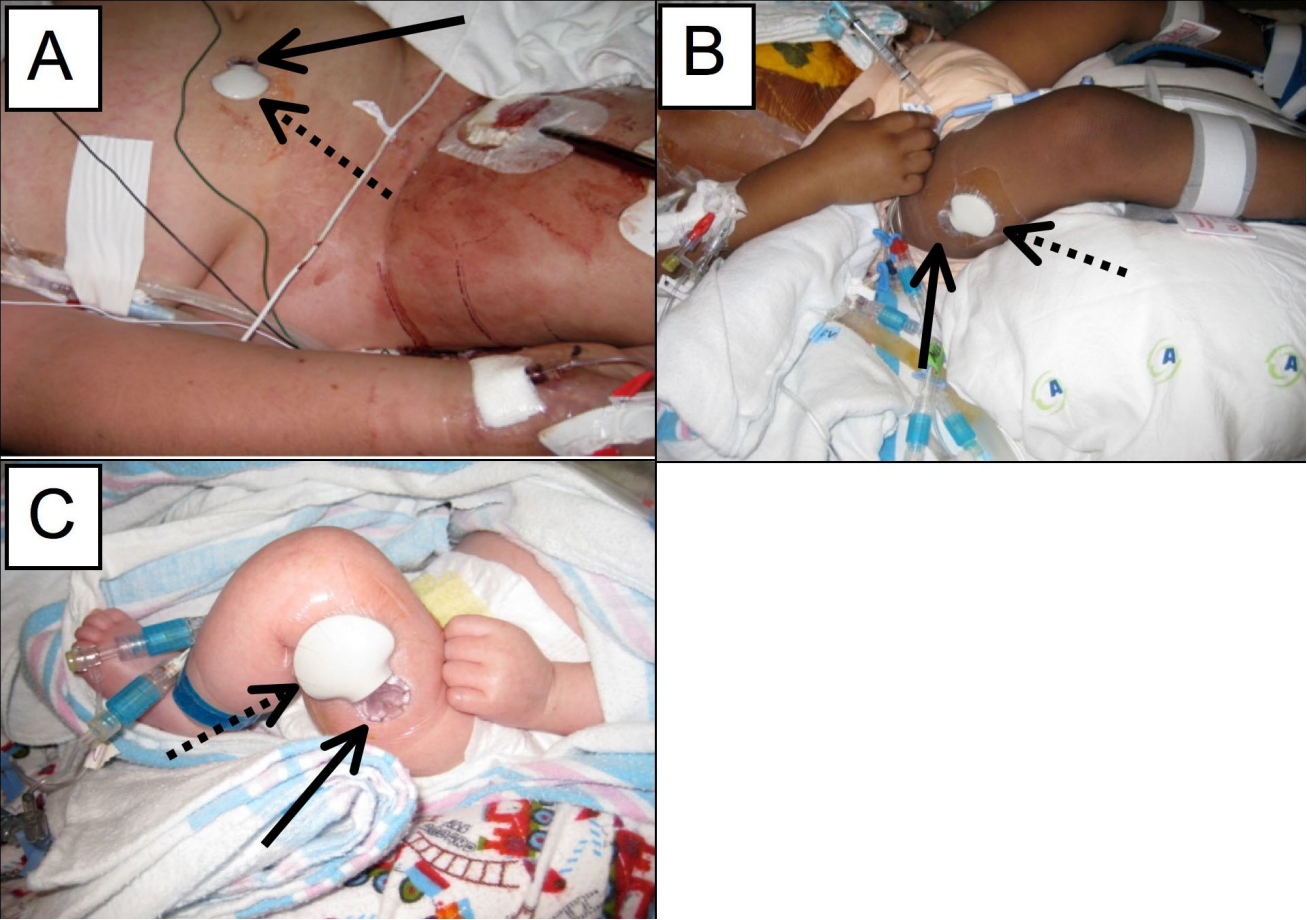
**Application of the CGM sensor and transmitter to pediatric patients**. The interstitial glucose sensor (solid arrow) was placed in the subdermal, interstitial space on the abdomen **(A) **or upper thigh **(B, C)**. The clam shell-appearing wireless transmitter (dotted line) was then attached. In A, the sensor was placed on a 10 year-old female with H1N1 and respiratory failure who subsequently required veno-venous ECMO (note the right femoral vein ECMO cannula and radial arterial catheter). Patient B depicts a three-year-old male trauma patient that had suffered a gunshot wound. Patient C was a six-week-old male status-post traumatic brain injury. The supplied clear dressing facilitates site observation for bleeding or reactions. Patients depicted in these photos were consented for photography.

We conducted a single-center, prospective, non-blinded, institutional review board-approved study. We enrolled 50 patients, ranging in age from 6 weeks to 16 years, admitted to our mixed medical/surgical or cardiac ICU at Children's Healthcare of Atlanta at Egleston who required mechanical ventilation and were at risk for developing critical illness hyperglycemia based on predefined risk factors. Patients with known type I diabetes mellitus were not considered for enrollment. Following informed consent, an area on the lower abdomen or thigh was cleaned with a chlorhexidine gluconate/isopropyl alcohol solution and the Medtronic Guardian^®^Real-Time Continuous Glucose Monitoring System sensor (Medtronic, Northridge, CA, USA) was placed via the manufacturer's recommended technique [13]. A wireless transmitter was attached to the sensor and covered with a supplied transparent dressing (Figure 1). Initial calibration of the CGM was performed using arterial, venous, or capillary point-of-care (POC) glucometry (iSTAT^®^, Abbot Laboratories, Princeton, NJ, USA) at two hours and six hours after the sensor placement. Subsequent calibrations occurred every 12 hours. The CGM recorded a glucose reading every five minutes. The sensor was replaced every five days during the study unless contraindicated, and it was removed when participants no longer required mechanical ventilation and/or vasoactive infusions. Although bedside nursing and physician teams were aware of enrollment, they did not assess CGM readings and no changes in patient management were based on values from the CGM. Audible alarms were set for CGM values of ≤ 70 mg/dL (3.9 mmol/L) and ≥ 200 mg/dL (11.1 mmol/L). With any alarm, the bedside nurses were instructed to obtain a POC glucose measurement, act accordingly to the POC value, and notify the study staff.

### Data acquisition and analysis

Demographic and clinical data were collected for all participants. CGM values were compared to POC and laboratory BG measurements that occurred at or within five minutes of CGM readings. A mixed model was performed to assess the relationship between CGM and BG measurements, as this accounts for the repeated measurements of glucose levels. CGM versus BG agreement was assessed using Clarke error grid analysis (Matlab^® ^R2009A, Natick, MA, USA) and Bland-Altman analysis. BG values used to calibrate the CGM were not used for comparison, but all other POC or laboratory BG measurements obtained during the study period were compared to CGM values. This included both BG measurements performed as part the patient's routine care and BG measurements obtained for a high or low glucose alarm from the CGM.

### Role of the funding source

This was an investigator-initiated study for which Medtronic^® ^(Northridge, CA, USA) donated CGMs, but provided no other funding or support. Internal institution funds were used to purchase the sensors for the GCM.

## Results

Fifty patients were enrolled, and a total of 89 sensors were used. There were 26 patients who had the sensor removed in less than five days, because they no longer met study criteria for critical illness hyperglycemia (that is, they no longer required mechanical ventilation, vasoactive medications, or continuous renal replacement therapy). One patient did not have the device in place long enough to record BG values, and two patients had dysfunctional sensors with no data recorded. The two patients with dysfunctional sensors were not significantly different in degree of illness or condition than the rest of the study patients. However, the sensors used on these two patients came from the same box. When a third patient started the study with a sensor from this box, it also did not work. When a sensor from a different box was used on this patient, it worked very well. Therefore, we concluded that this box of sensors was defective, and it was not used during the rest of the study.

Of the 47 patients with accessible data, the mean age was 4.3 years (range 6 weeks to 16 years-old) and 31 (66%) were male. Twenty-nine (62%) were medical pediatric ICU patients, 8 (17%) were surgical pediatric ICU patients (including general surgery, trauma surgery, neurosurgery, and otolaryngology), and 10 (21%) were cardiac surgery patients. Six (13%) had traumatic brain injury or intracranial hemorrhage. All patients required mechanical ventilation, and 30 (63.8%) required vasopressor or inotropic infusions. Twenty (42.6%) developed critical illness hyperglycemia, defined as persistent BG levels >140 mg/dL (7.7 mmol/L), and received insulin via our published pediatric-specific hyperglycemia protocol [14,15]. Six (12.8%) required continuous renal replacement therapy (CRRT), and three (6%) developed the need for veno-venous extracorporeal membrane oxygenation (ECMO). Participants had indicators consistent with high level of illness severity, including a mean ICU length of stay (LOS) of 15 days. A total of 17 (36%) patients had pediatric logistic organ dysfunction (PELOD) scores ≥12 (Table 1).

**Table 1:** Patient demographics

Category	N	Glucose comparisons	Age (yr)	Weight (kg)	% Female (N)	Ethnicity (N)	ICU LOS (days)	% CIH (N)	CGMdays	Pearson's correlation coefficient
All	47	1555	4.3 (0.01 to 16)	20.6 (2.4 to 87)	34% (16)	AA 1.1% (24) H 8.5% (4) C 36.2% (17)	15 (2 to 102)	79% (34)	6.1 (1 to 18)	.68
**A. Primary diagnosis**										
Medical	29	895	4.6 (0.1 to 14)	22.5 (2.9 to 87)	41.4% (12)	AA 51.7% (15) H 10.3% (3) C 31% (9)	13.4 (2 to 41)	66% (19)	6.2 (1 to 15)	.71
Cardiac surgery	10	222	1.2 (0.01 to 7)	7.5 (2.4 to 20.2)	40% (4)	AA 30% (3) H 10% (1) C 60% (6)	11 (2 to 44)	70% (7)	3.2 (1 to 7)	.81
Surgery/other	8	438	7.1 (1.9 to 16)	30.2 (12.7 to 59)	0% (0)	AA 75% (6) H 0% (0) C 25% [39]	25.6 (4 to 102)	100% (8)	9.4 (1 to 18)	.50
TBI/ICH	6	113	5.3 (0.1 to 14)	21.4 (3.3 to 50)	16.7% (1)	AA 33.3% [39] H 0% (0) C 66.7% (4)	10.2 (6 to 15)	14% [39]	5.5 (2 to 14)	.59
**B. Support**										
VPI/Inotropes	30	1184	4.4 (0.01 to 16)	19 (2.4 to 87)	43.3% (13)	AA 50% (15) H 10% (3) C 33.3% (10)	17.6 (2 to 102)	80% (24)	6.5 (1 to 18)	.73
IV Steroids	32	1321	4 (0.01 to 16)	20 (2.4 to 87)	31.3% (10)	AA 65.6% (21) H 6.3% [39] C 25% (8)	17.4 (2 to 102)	81% (26)	6.6 (1 to 18)	.69
CVVH	6	495	6.8 (1.3 to 16)	34.7 (8 to 87)	50% (3)	AA 83.3% (5) H 0% (0) C 16.7% (1)	34.5 (12 to 102)	83% (5)	10.3 (4 to 18)	.76
ECMO	3	216	5.8 (1.3 to 16)	39.7 (10 to 87)	33.3% (1)	AA 66.7% [39] H 0% (0) C 33.3% (1)	25 (12 to 38)	100% (3)	12 (8 to 18)	.57
**C. CIH**										
No	13	96	2 (0.04 to 9)	12.8 (2.4 to 55)	30.8% (4)	AA 53.8% (7) H 23.1% (3) C 23.1% (3)	10.1 (2 to 25)	0% (0)	3.3 (1 to 7)	.68
Yes - without insulin therapy	14	324	1.7 (0.01 to 14)	12.2 (2.9 to 74)	35.7% (5)	AA 35.7% (5) H 0% (0) C 50% (7)	14.5 (2 to 41)	100% (14)	6.1 (1 to 13)	.71
Yes - with insulin therapy	20	1135	7.5 (0.01 to 16)	31.7 (3.5 to 87)	35% (7)	AA 60% (12) H 5% (1) C 35% (7)	18.6 (2 to 102)	100% (20)	7.9 (1 to 18)	.67
**D. Age**										
Less than one years	18	374	0.3 (0.01 to 0.8)	5.3 (2.4 to 9.2)	38.9% (7)	AA 38.9% (7) H 11.1% [39] C 44.4% (8)	15.4 (2 to 44)	56% (10)	5.3 (1 to 12)	.75
One to five years	13	416	2.3 (1.2 to 4)	13.7 (10 to 20)	23.1% (3)	AA 61.5% (8) H 7.7% (1) C 23.1% (3)	10.3 (2 to 33)	77% (10)	5.5 (1 to 13)	.64
6 to 10 years	9	488	7.6 (6 to 10)	34.9 (20.2 to 87)	44.4% (4)	AA 55.6% (5) H 11.1% (1) C 33.3% (3)	15.2 (2 to 38)	78% (7)	8 (1 to 18)	.73
More than 10 years	7	277	14 (13 to 16)	54.6 (32.4 to 74)	28.6% [39]	AA 57.1% (4) H 0% (0) C 42.9% (3)	22.4 (2 to 102)	100% (7)	6.7 (1 to 14)	.55
**E. PELOD**										
<12	30	604	4.1 (0.01 to 14)	20.3 (2.4 to 74)	30% (30)	AA 46.7% (14) H 13.3% (4) C 33.3% (10)	11.8 (2 to 41)	63% (19)	4.9 (1 to 18)	.69
≥12	17	951	4.6 (0.01 to 16)	21.3 (3.3 to 87)	41.2% (7)	AA 58.8% (10) H 0% (0) C 41.2% (7)	20.6 (3 to 102)	88% (15)	8.2 (1 to 15)	.68
**F. ICU LOS**										
Less than three days	5	45	5 (0.2 to 14)	25.2 (6.2 to 74)	41.2% (7)	AA 60% (3) H 20% (1) C 20% (1)	2 (2 to 2)	60% (3)	1.4 (1 to 2)	.74
Three to seven days	12	139	3.8 (0.1 to 14)	17.9 (3.3 to 67.8)	8.3% (1)	AA 33.3% (4) H 16.7% [39] C 41.7% (5)	5 (3 to 7)	58% (7)	2.2 (1 to 5)	.85
More than seven days	30	1371	4.3 (0.01 to 16)	21 (2.4 to 87)	43.3% (13)	AA 56.7% (17) H 3.3% (1) C 36.7% (11)	21.2 (8 to 102)	80% (24)	8.4 (2 to 18)	.66

Numbers indicate mean values unless otherwise indicated. Numbers in parentheses ( ) represent number or range. VPI, vasopressor infusions; CVVH, continuous veno-venous hemofiltration; AA, African American; C, Caucasian; CIH, critical illness hyperglycemia; H, Hispanic; LOS, length of stay.

There were a total of 142 episodes of CGM readings <40 mg/dL (2.2 mmol/L). Readings <40 mg/dL (2.2 mmol/L) from the CGM accounted for 0.2% of the total 64,315 CGM readings. All of the CGM readings of <40 mg/dL (2.2 mmol/L) took place in just five different patients. When checked against BGs, these CGM readings of <40 mg/dL (2.2 mmol/L) were shown to be falsely low. The lowest BG drawn during an episode in which the CGM reading was <40 mg/dL (2.2 mmol/L) was 71 mg/dL (3.9 mmol/L). All of these episodes of falsely low CGM readings corrected with sensor recalibration or replacement. There were no episodes of hypoglycemia of <40 mg/dl (2.2 mmol/L) in any of the POC or laboratory BGs during the entire study.

**Figure 2:**
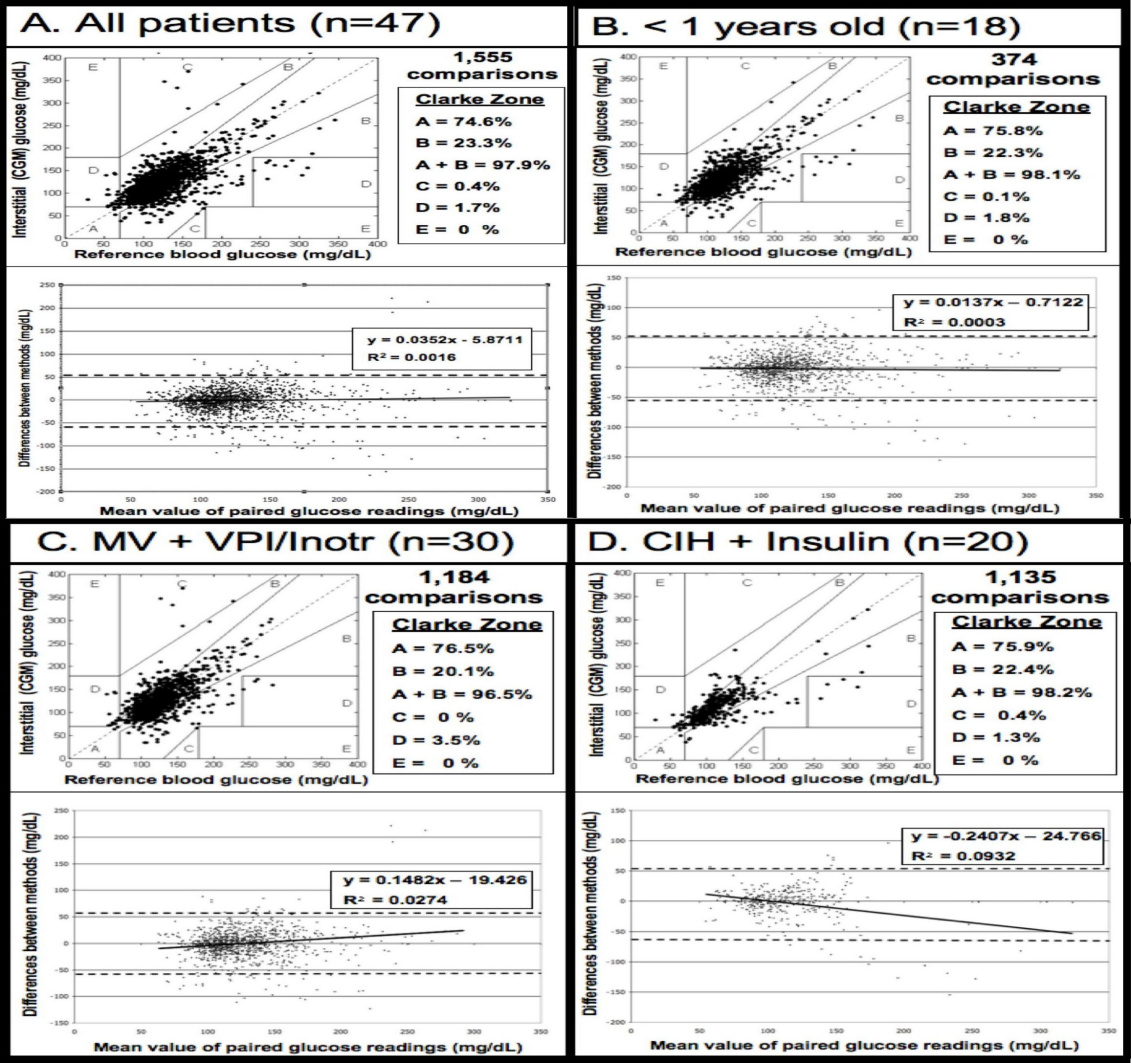
**Clarke error grid and Bland-Altman agreement analysis of paired BG readings**. A total of 1,555 CGM values from 47 pediatric ICU patients were compared for agreement with BG measurements **(A)**. In Clarke plots (upper panel), the routine POC readings (X-axis) were compared to corresponding CGM values (Y-axis). Readings in Zone A differ by ≤20%, whereas those in Zone B differ by >20% but do not impact management. The Zone A + B composite is considered "clinically acceptable" correlation. Zone C values would lead to therapeutic overcorrection and Zone D readings would not trigger intervention, although warranted. Zone E values would result in treatment aggravating a hypo- or hyperglycemic state. In Bland-Altman analysis (bottom panel), the mean of the paired readings is on the X-axis, and the difference is on the Y-axis. The broken line represents the cutoff of two standard deviations from the mean difference. In addition to the R^2^, the solid line with the indicated slope is shown and represents the correlation coefficient. Clarke and Bland-Altman plots are shown for patient subpopulations less than one-year-old **(B)**, those who received vasopressor or inotropic support **(C)**, and those who developed critical illness hyperglycemia and were managed with insulin using our protocol **(D)**.

A total of 1,555 paired CGM and BG measurements were analyzed using Clarke error grid and Bland-Altman analysis (Figure 2). For the 1,555 paired CGM and BG measurements, there is a statistically significant linear relationship between CGM values and BG (*P *<.0001). A one unit increase in CGM increases BG by an average of .6537.

With Clarke analysis, readings in Zone A differ by ≤20%, whereas those in Zone B differ by >20% but do not impact management. Readings in Zone A and B are considered to have clinically acceptable correlation. Zone C values would lead to therapeutic overcorrection and Zone D readings would not trigger intervention, although warranted. Zone E values would result in treatment aggravating a hypo- or hyperglycemic state. With Clarke error grid analysis of all patients, 74.6% of readings were in Zone A and 23.3% of readings were in Zone B, equating to 97.9% of all readings with clinically acceptable correlation (Zone A + B). A total of 2.1% of readings were in Zones C + D, and no readings were in Zone E. Pearson's correlation coefficient for all comparisons was 0.68. In Bland-Altman agreement analysis, approximately 95% of all CGM values were +/- 58 mg/dL (3.2 mmol/L) from the mean difference of -1.5 mg/dL (0.08 mmol/L) between the CGM and BG values. The mean absolute relative difference (MARD) between CGM and BG values was 15.3%. The MARD gives the average absolute difference between two methods of measurement.

**Figure 3:**
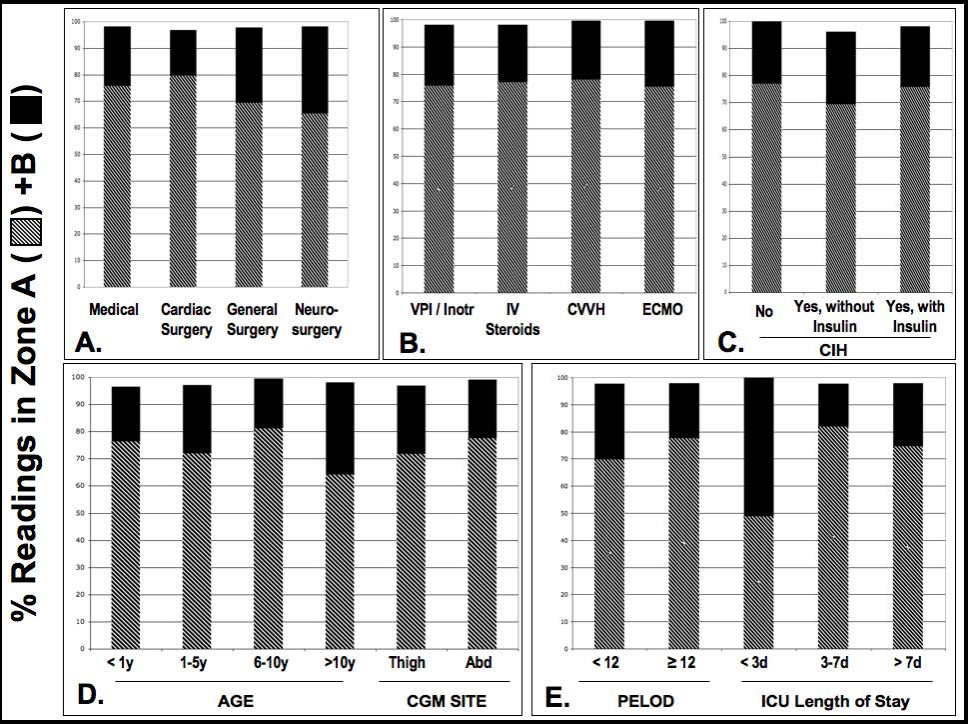
**Percentage of clinically acceptable paired glucose readings in patient subpopulations**. Patients were grouped based on diagnosis **(A)**, age or sensor site placement **(B)**, support measures in addition to mechanical ventilation **(C)**, development and/or treatment of critical illness hyperglycemia **(D)**, and severity of illness indicators **(E)**. See Table 1 for corresponding subpopulation details. Clarke analysis was conducted and shown as the percent of readings in Zone A and B. As is standard, we defined "clinically acceptable" correlations as the sum of Zone A and B.

We compared BG and CGM glucose values in a variety of subpopulations to investigate whether patient age, diagnosis, support measures, or illness severity influenced CGM function. We found a high correlation of glucose values (that is, Clark Zone A + B >95%) in all age ranges, including our youngest (<12 months old), and in all diagnostic categories. Sensor placement (thigh vs. abdomen) did not impact reading accuracy. There was high correlation in children who required support measures in addition to mechanical ventilation, including vasopressors, inotropes, CRRT, and/or ECMO (Figures 2 and 3). Clinically acceptable agreement was not obviously different in those with low or high illness severity scores or according to ICU LOS.

We also investigated whether CGM values correlated with BG values in patients who developed critical illness hyperglycemia and received insulin to maintain BG in the 80-140 mg/dL (4.4 to 7.7 mmol/L) range [5,12,14,15]. We found a high correlation between BG and CGM readings (98.2% in Zone A + B) in patients who required an insulin infusion for critical illness hyperglycemia (Figure 2D).

No patient with CGM sensors in our study developed a site infection, reaction, or bleeding. The insertion of sensors was well tolerated with no obvious discomfort, albeit all patients were receiving sedation and/or analgesia, because they were intubated and mechanically ventilated. There was no obvious interference with the CGM from ICU monitors or electronic support devices. The sensors and transmitters are not MRI compatible, and for study subjects who required such imaging, the sensors and transmitters were removed and then replaced after the exam was complete.

**Figure 4:**
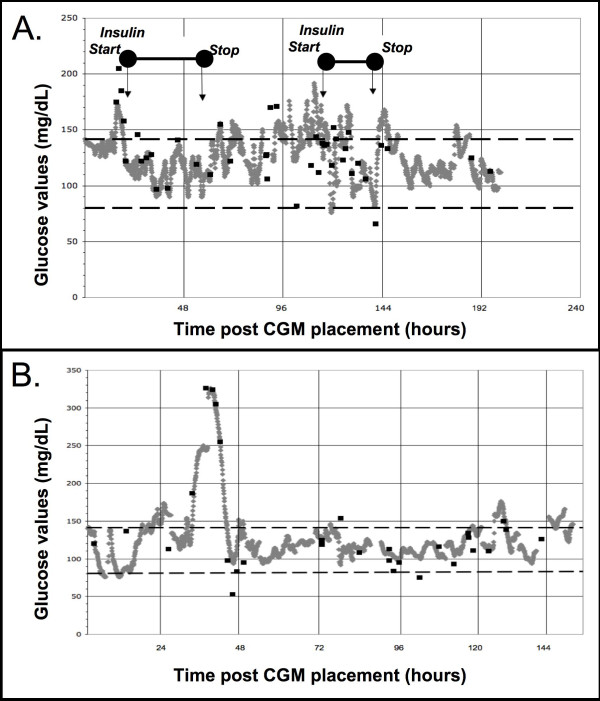
**Temporal plot comparing CGM readings and BG values**. With software provided with the CGM, CGM values and BG values were plotted over time. CGM values (gray dots that at times morph into a line) are acquired approximately every five minutes, and BG values (black squares) sporadically per regular care. We define persistent BG >140 mg/dL (7.7 mmol/L) as critical illness hyperglycemia, and have traditionally managed those more than six months old with insulin infusions to a target BG of 80 to 140 mg/dL (4.4 to 7.7 mmol/L) (black dashed lines). The top plot **(A) **represents data from a 13 year-old male with traumatic brain injury requiring mechanical ventilation and norepinephrine to maintain his cerebral perfusion pressure. The arrows indicate when insulin was started and stopped per our protocol to manage his hyperglycemia. The data in **B **is from a five-month-old male with Trisomy 21, RSV, pulmonary hypertension, and refractory hypoxemia requiring high frequency oscillatory ventilation, inhaled nitric oxide, milrinone, and an epinephrine infusion. At the time of this study, this patient was below our typical age threshold for protocolized hyperglycemic management, and he was not treated with insulin.

Using software provided with the CGM, we overlaid CGM and BG readings. Shown in Figure 4 are two examples; one of a patient who developed critical illness hyperglycemia and was managed with insulin and another of a patient who developed hyperglycemia but did not receive insulin due to age restrictions.

## Discussion

Our study demonstrates that commercially available, interstitial CGMs correlate closely with BG measurements in critically ill children. Glycemic control in critically ill adults improves outcomes in a number of studies from adult medical, surgical, and mixed ICUs, and is recommended as standard practice by many medical advisory committees [1,2,8,9,16]. A recent report by Vlasselaers *et al. *also demonstrated clinical benefits of strict glycemic control in a pediatric medical-surgical ICU [4]. However, one issue that has plagued many glycemic control trials in adults and children has been unacceptably high rates of iatrogenic hypoglycemia in strict control groups. Although the effect of hypoglycemia on outcomes is unclear, these concerns have resulted in a lack of adoption of routine glycemic management by some ICU practitioners (for example, those in pediatric critical care).

Advocates and critics of glycemic control agree that a reliable means to continuously sample and report glucose levels will assist in understanding the effects of ICU hyperglycemia and its management. Such technology would improve both the consistency and safety of glycemic control approaches. Notably, in discussing the high rates of hypoglycemia despite outcome benefit in their recently published RCT in pediatric critical care, Vlasselaers *et al. *state that "...for future studies, an accurate continuous blood glucose sensor for use in the PICU...would be preferable to keep the risk of hypoglycaemia to a minimum [4]."

CGMs, which regularly assess interstitial glucose levels, have been developed, approved, and marketed for outpatient diabetes management. Although ICU practitioners support the concept of utilizing such devices, many have dismissed the potential use of such technology in critical care settings. Their argument is that there may be an inaccuracy of interstitial glucose values compared to blood levels in patients with edema, poor perfusion, or in those requiring vasoactive infusions. Despite these theoretical drawbacks of CGM and interstitial glucose assessments, little evidence exists to substantiate these concerns. There is now a small cohort of limited studies of approximately 160 adult and pediatric ICU patients suggesting that there is a reasonable correlation between CGM and BG measurements [17-27].

We report one of the largest studies to date evaluating a Food and Drug Administration (FDA) approved, commercially available CGM in a critical care setting. We assessed patients that our previous studies have proved to be at increased risk for critical illness hyperglycemia [12,14,15,28,29]. Currently, there is debate on which is the gold standard blood supply (that is, arterial vs. venous) to assess BG. In daily management, ICUs likely use the most convenient and accessible vascular bed for blood glucose determination and glycemic management, including capillary sampling. Our study may provide generalizable and relevant data for practitioners and staff who may use different sources for BG measurements.

In all patients and in subpopulation analysis based on age, diagnosis, and ICU support measures, we found a high correlation between CGM readings and standard BG measurements per Clarke error grid and Bland-Altman analysis. Although some vasoactive medications can result in peripheral vasoconstriction, this did not affect the accuracy of interstitial readings in our study. Correlation was also strong in those requiring CRRT and/or ECMO, despite significant edema and vascular leak syndrome in these patients. A 2005 report demonstrated that a CGM device attached to an ECMO circuit providing support for pediatric patients, could supply reasonable glucose readings [30]. Our study appears to be the first to evaluate CGMs on patients who required ECMO, with the important note that sensors were placed on patients before they were placed on ECMO.

Patients who were more severely ill according to traditional illness severity indicators (PELOD scores, ICU lengths of stay) had excellent correlation between CGM and standard readings. In fact, those with an ICU stay of less than three days had the lowest Clark Zone A correlation in our analysis. This may be due to the fact that the precision of CGMs may improve with time after insertion. It is interesting to note that one study that found a poor correlation of CGMs in hospitalized patients, sensors were in place for a maximum of three days [27]. In addition, while some have questioned the utility of such devices in very small pediatric patients, we found excellent correlation in patients <12 months of age. Also, we found excellent correlation in pediatric ICU patients who were hyperglycemic and managed with an insulin infusion.

A number of CGM devices have been approved for outpatient use in children and adults [31-33]. FDA approval applications have been supported by 90 to 99% Clarke A + B correlation between CGM and standard BG readings, and 1.6 to 10% of clinically inaccurate readings (Zone C + D + E). Such applications cite studies evaluating approximately 20 to 137 patients [34-36]. Findings from our study of 47 participants, with Zone A + B of 97.9% and Zone B + C + D of 2.1%, are consistent with criteria used in other venues to determine acceptable accuracy. In addition, we found no untoward effects of CGM use. Although critically ill patients often have coagulopathies, we found no evidence of bleeding with device use. Of note, sensors were not inserted when a known severe coagulopathy was present. In addition, we also found no site infections, reactions, or significant discomfort noted with sensor placement or use.

Our data provide some of the most robust evidence to date that these devices can be used safely and provide clinically meaningful information, even in the smallest and most critically ill pediatric patients. In conjunction with other CGM studies in ICU settings, it may be reasonable to consider incorporating the use of such devices into critical care practice, albeit with some important limitations. We found that there is a significant time and educational component needed to learn to insert, calibrate and assess these devices. We also frequently kept sensors in place for longer than the FDA-recommended time. This sensor is currently approved for 72 hours of use in the outpatient setting, and there are no guidelines for their use in the ICU setting. However, there are reports of these sensors being accurate and safe for longer than the 72-hour period [37]. Medtronic has recently completed a study testing the use of this sensor for six days in an inpatient setting, and these results are pending [38]. Although our findings demonstrate a strong correlation even with extended use, we did not make any clinical management decisions based on CGM readings. Only after further study in clinical trials should such devices be used to assist in titrating insulin during hyperglycemia management in critical care settings. Active diagnosis and/or management of glucose disturbances in ICU settings should only occur with time-tested BG measurements.

The current state of evidence in this field may support the use of such devices to trend patient BGs, and setting high and low alarms to trigger standard BG assessment may assist in improving the efficacy and safety of glycemic control approaches. We experienced very few hypoglycemic episodes in this study, and thus the accuracy of CGM in low-normal or hypoglycemic ranges is unclear. Anecdotally, although our staff was essentially blind to the readings of these monitors, there were times when the "low" BG alarm was triggered, prompting our staff to perform a routine BG check. In one notable case, a hyperglycemic patient treated with insulin had her intravenous fluids switched from a high to low dextrose-containing fluid without a change in insulin dosing. The patient was not due to receive a routine BG check, but after the CGM alarmed, a POC BG was performed and confirmed a low reading. The IV fluid discrepancy was noted, and the insulin infusion was changed. As such, the most important utility of CGMs at this time may be to trigger standard BG checks to improve the safety of glycemic control.

Data from CGM devices will likely add to the understanding of glycemic control in the critically ill patient. As shown in Figure 4, obtaining daily glucose trends via CGM may help to identify the percent of time spent outside of goal target ranges. Accurate, real-time assessment of area-under-the-curve metrics can be obtained from CGM devices. This may provide more meaningful data compared to approaches currently used to define and compare glycemic control.

## Conclusions

Critically ill children encompass one of the widest spectrums of patients, in terms of size and condition, in any ICU setting. The severity of illness and support measures of patients in this study were considerable. It may be reasonable to consider that if CGMs function well in our study population, they will likely function well in most other ICU populations, whether pediatric, adult, medical, or surgical. Yet, there will likely be scenarios where CGM performance will be sub-standard, and careful assessment during its use is required. Despite the proven benefits of glycemic control in many critical care settings, there are recognized risks of this approach and more studies are needed to better define optimal BG target ranges in the spectrum of ICU patients.

Our study adds to the body of evidence that refutes the theoretical concerns regarding the use of CGM devices in the ICU setting. There is no doubt that future technologies will help advance the field of glycemic control in critical illness. The evidence may now support the use of currently available CGMs to assist with the safety and efficacy of glycemic control in critical illness. Further study and application for clinical approval of CGM use in ICU settings should be paramount in the international effort to better understand critical illness hyperglycemia.

## Key messages

• Hyperglycemia, hypoglycemia and glucose variability are associated with poor outcomes in critical illness. However, recent studies on the control of critical illness hyperglycemia have shown unacceptably high rates of iatrogenic hypoglycemia.

• This study demonstrates that interstitial CGMs have a high degree of correlation with BG levels in a wide variety of critically ill children.

• Our findings suggest that with further examination, existing CGM technology may be a useful adjunct in the detection and management of glucose disorders in critically ill children.

## Abbreviations

BG: blood glucose; CGM: continuous glucose monitor; CRRT: continuous renal replacement therapy; ECMO: extracorporeal membrane oxygenation; FDA: Food and Drug Administration; LOS: length of stay; MARD: mean absolute relative difference; PELOD: pediatric logistic organ dysfunction; POC: point-of-care; RCT: randomized controlled trial

## Competing interests

The authors declare that they have no competing interests.

## Authors' contributions

BCB, CMP, KOM and MRR contributed equally to the design, acquisition of data, and analysis of data for this study. All of the authors were involved in drafting the manuscript and revising it critically for important intellectual content. They all gave final approval of the version to be published.
